# Effects of parity, blood progesterone, and non-steroidal anti-inflammatory treatment on the dynamics of the uterine microbiota of healthy postpartum dairy cows

**DOI:** 10.1371/journal.pone.0233943

**Published:** 2021-02-19

**Authors:** O. Bogado Pascottini, J. F. W. Spricigo, S. J. Van Schyndel, B. Mion, J. Rousseau, J. S. Weese, S. J. LeBlanc

**Affiliations:** 1 Population Medicine, Ontario Veterinary College, University of Guelph, Guelph, ON, Canada; 2 Department of Animal Biosciences, University of Guelph, Guelph, ON, Canada; 3 Department of Pathobiology, Ontario Veterinary College, University of Guelph, Guelph, ON, Canada; Michigan State University, UNITED STATES

## Abstract

This study evaluated the effects of treatment with meloxicam (a non-steroidal anti-inflammatory drug), parity, and blood progesterone concentration on the dynamics of the uterine microbiota of 16 clinically healthy postpartum dairy cows. Seven primiparous and 9 multiparous postpartum Holstein cows either received meloxicam (0.5 mg/kg SC, n = 7 cows) once daily for 4 days (10 to 13 days in milk (DIM)) or were untreated (n = 9 cows). Endometrial cytology samples were collected by cytobrush at 10, 21, and 35 DIM, from which the microbiota analysis was conducted using 16S rRNA gene sequence analysis. A radioimmunoassay was used to measure progesterone concentration in blood serum samples at 35 DIM and cows were classified as ˃ 1 ng/mL (n = 10) or ≤ 1 ng/mL (n = 6). Alpha diversity for bacterial genera (Chao1, Shannon-Weiner, and Camargo’s evenness indices) were not affected by DIM, meloxicam treatment, parity, or progesterone category. For beta diversity (genera level), principal coordinate analysis (Bray-Curtis) showed differences in microbiota between parity groups. At the phylum level, the relative abundance of Actinobacteria was greater in primiparous than multiparous cows. At the genus level, there was lesser relative abundance of *Bifidobacterium*, *Lactobacillus*, *Neisseriaceae*, *Paracoccus*, *Staphylococcus*, and *Streptococcus* and greater relative abundance of *Bacillus* and *Fusobacterium* in primiparous than multiparous cows. Bray-Curtis dissimilarity did not differ by DIM at sampling, meloxicam treatment, or progesterone category at 35 DIM. In conclusion, uterine bacterial composition was not different at 10, 21, or 35 DIM, and meloxicam treatment or progesterone category did not affect the uterine microbiota in clinically healthy postpartum dairy cows. Primiparous cows presented a different composition of uterine bacteria than multiparous cows. The differences in microbiota associated with parity might be attributable to changes that occur consequent to the first calving, but this hypothesis should be investigated further.

## Introduction

After parturition, the endometrial epithelium and caruncles are sloughed and the uterus of most cows is exposed to potentially pathogenic bacteria [1, 2]. Up to half of dairy cows experience at least one form of reproductive tract inflammatory disease within 35 days in milk (DIM) [3]. This takes the form of systemic illness with fetid uterine discharge before 14 DIM (metritis), or localized infection of the uterus and/or cervix (diagnosed as purulent vaginal discharge (PVD) around 5 weeks postpartum), or localized inflammation diagnosed by cytology (without PVD) referred to as subclinical endometritis (SCE). The two former conditions are clearly associated with bacterial pathogens: metritis predominantly with Gram-negative anaerobes (e.g., *Fusobacterium* and *Porphorymonas*) [4], and PVD with *Trueperella pyogenes* [5]. SCE has not been associated with changes in the uterine microbiota, in comparison to healthy cows [5, 6]. The point at which uterine inflammation and uterine microbiota shifts from physiologic to pathologic and the determinants of these processes are only partially understood [7]. Questions remain about which bacteria may be critical in pathogenesis, and how the interplay between bacterial infection and host response lead to or avoid disease [2].

Studies using conventional bacteriology methods rely on the ability to culture live bacteria that grow under controlled conditions in the lab, whereas genetic sequencing technologies allow identification of a fuller range of uterine bacteria [8]. The advent of culture-independent methods such as 16S ribosomal RNA gene sequencing has advanced knowledge about bacterial communities in the reproductive tract of cows [9]. Understanding the dynamics of the uterine microbiota and the factors that affect the microbiota may provide deeper insights into the pathophysiology of uterine disease. Genial tract decompartmentalization and dysbiosis within the first week postpartum has been associated with metritis and endometritis [10, 11]. However, not much is known about the dynamics of uterine microbiota in healthy postpartum cows and the factors that may affect its composition.

Many physiological conditions such as resident uterine and vaginal microbiota, time relative to calving, degree of metabolic stress, and concentrations of sex hormones or other changes inherent in the estrus cycle may play a role in the uterine bacterial composition of healthy postpartum cows. Among these physiological conditions, the potential effect of parity (primiparous versus multiparous) on the uterine microbiota is not clear. The size and position of the uterus in multiparous cows (more pendulous), may affect the uterine microbiota in the early postpartum period. Moreover, the baseline microbiota before pregnancy or before calving may differ between nulliparous and parous animals. Early ovulation may also have an effect on the uterine microbiota. It is generally accepted that high progesterone concentrations suppress the immune response [12], but it is challenging to establish uterine infections while estrogens are dominant [12, 13]. However, not much is known about the role of circulating progesterone concentration on the uterine microbiota in healthy postpartum cows.

In a previous study, we showed that treatment with the non-steroidal anti-inflammatory drug (NSAID) meloxicam from 10 to 13 DIM transiently reduced haptoglobin (a marker of systemic inflammation) and improved indicators of energy metabolism (greater insulin-like growth factor-1 and glucose, and lesser β-hydroxybutyrate), and modestly increased circulating polymorphonuclear (PMN) cell function [14]. Furthermore, antibacterial properties of some NSAID were recently discovered [15]. Diclofenac, aspirin, and etodolac could prevent biofilm formation at normal doses used during anti-inflammatory therapy [16]. However, the mechanisms of possible antimicrobial activity of NSAID are mostly unclear. For the present study, the hypothesis was that either metabolic changes or a direct effect induced by meloxicam would affect the uterine microbiota. Thus, our objective was to evaluate the effects of meloxicam on the uterine microbiota of clinically healthy postpartum dairy cows. We also aimed to study the effects of DIM at sampling, parity, and blood progesterone concentration on the dynamics of the uterine microbiota at 10, 21, and 35 DIM.

## Materials and methods

### Ethics approval

Animal handling procedures, sampling, and treatment were approved by the University of Guelph Animal Care Committee (Animal Utilization Protocol #3852). Cows were managed according to the code of practice of the National Farm Animal Care Council.

### Study design

This study derives from a larger experiment on postpartum NSAID treatment [14] that was conducted from April to August 2018 at the University of Guelph Livestock Research and Innovation Centre, Dairy Facility (Elora, ON, Canada). For that study, 20 out of 42 Holstein cows received subcutaneous injections of meloxicam (0.5 mg/kg of body weight; Metacam, Boehringer Ingelheim Canada Ltd., Burlington, ON, Canada) once daily on 10, 11, 12 and 13 DIM (the rationale for this meloxicam dosing is described by Pascottini et al. [14]. In a subsequent experiment using 21 cows from the underlying study, we compared the uterine bacterial composition of postpartum cows diagnosed with SCE (absence of mucopurulent vaginal discharge but with ˃ 5% endometrial polymorphonuclear neutrophils (PMN); n  =  8), clinical endometritis (mucopurulent or purulent vaginal discharge with ˃ 5% endometrial PMN; n  =  5), or healthy (neither SCE nor CE; n = 8) in the fifth week postpartum [6]. Uterine bacterial composition was not different between healthy and SCE cows, but uterine microbiota differed in cows with clinical endometritis [6]. For the present study, the remaining 16 cows without clinical endometritis were classified as primiparous (n = 7) or multiparous (n = 9), treated with meloxicam (n = 7) or control (n = 9), and with high (˃ 1 ng/mL; n = 10) or low (≤ 1 ng/mL or absence of corpus luteum; n = 6) progesterone serum concentration at 35 DIM to compare their uterine bacterial composition.

### Management

Cows calved in individual box stalls and were moved to free-stall pens 5 days after calving. Cows were fed *ad libitum* from individually assigned automated feed bins (Insentec B.V., Marknesse, the Netherlands), and were milked twice daily in a rotary parlor. Diet and milk production data are reported in Pascottini et al. [14]. Only cows considered clinically healthy from calving to 35 DIM were included (unassisted calving and absence of retained placenta, metritis, or other clinical disease (including PVD) before or during the study period).

### Blood sampling and serum progesterone analysis

Blood samples were collected by coccygeal venipuncture into vacuum tubes without anticoagulant (BD Vacutainer Precision Glide, Becton Dickinson, Franklin Lakes, NJ) at 35 DIM. All blood samples were allowed to clot, centrifuged at 1,500 × g for 15 min and serum was stored in aliquots at -20°C. Progesterone was measured using the Progesterone Double Antibody RIA Kit (MPBiomedicals, Inc., Costa Mesa, CA, USA) according to the manufacturer’s instructions. Briefly, 100 μL of each standard, serum sample, and control were added into their respective anti-progesterone coated tubes. Then, 1 mL of progesterone I-125 was added to all tubes and vortexed briefly. Tubes were then incubated in a 37°C-water bath for 120 min. The contents of the tubes were decanted, and tubes were counted in a gamma counter calibrated for I-125 (Biodex Medical Systems, Inc.). The lower limit of detection was 0.1 ng/ml, and the intra-assay coefficient of variation was 14.3%. Samples were analyzed in the same batch.

### Cytology collection

Endometrial cytobrush samples were collected at 10, 21, and 35 DIM. Briefly, after cleansing the perineal area of the cow with antibacterial soap and water, 70% isopropyl alcohol was sprayed and dried thoroughly with paper towels. A sterile cytobrush rod (covered by a sterile sanitary sheath) was introduced into the vagina and guided through the cervix via rectal palpation. Once the tip of the rod reached the uterine body, the sanitary sheath was pulled back, the cytobrush was exposed from the rod and rotated against the dorsal wall of the endometrium (*corpus uteri* region) with gentle pressure from the index finger through the rectum. The cytobrush was retracted into the rod and removed from the vagina. Once outside the genital tract, the cytobrush was gently rolled onto a sterilized microscope slide. The cytobrush tip was then cut with sterilized scissors, inserted into a sterile 2 mL cryovial, and stored at -80°C within 15 min. After cytobrush sampling, vaginal discharge was evaluated using the Metricheck device (Simcrotech, Hamilton, New Zealand) and scored as 0 = clear mucus, 1 = mucus with flecks of pus, 2 = mucopurulent discharge (≤ 50% pus), and 3 = purulent discharge (> 50% pus) [17]. Cytobrush microscope slides were stained using May-Grunwald-Giemsa stain, 300 cells were counted per slide in multiple fields, and the proportion of PMN cells to epithelial cells (PMN%) was determined.

### DNA extraction

DNA was extracted using the QIAamp Microbiome kit (Qiagen Inc.; Toronto, ON, Canada) following the instructions of the manufacturer. This kit isolates bacterial DNA from swabs and body fluids and effectively depletes the host DNA. It uses both chemical and mechanical methods to achieve cell lysis. The first reagent of the extraction procedure was added directly into the 2 mL cryovial containing the cytobrush tip. Six sterile cytobrush tips were exposed to the environment of the barn (in the air for 5 s), placed in 2 mL cryovials, and stored at -80°C within 5 min. These samples were the negative controls in the sequencing. A mock community (ATCC MSA-1002, Cedarlane Labs, Burlington, ON, Canada) containing twenty known strains was used for quality control. The DNA concentration was measured using a NanoDrop ND-1000 uv-vis spectrophotometer (Thermo Fisher Scientific; Wilmington DE, USA) based on the absorbance at 260 nm and using the Beer-Lambert equation. The DNA quality was assessed by using the 260/280 nm ratio. The average 260/280 ratio was 1.93 with a range of 1.5 to 2.2.

### 16S rRNA gene amplification and sequencing

PCR amplification of the V4 hypervariable region of the 16S rRNA gene was performed using the forward primer 515F-mod and reverse primer 806R-mod [18]. The forward and reverse primers were designed to contain an Illumina® overhang adapter sequence (Illumina®; San Diego, CA, USA) in order to anneal them to primers containing the Illumina® adaptors plus the 8 bp identifier indices. The following PCR conditions were used: 3 minutes at 94°C for denaturing, followed by 30 cycles of 45 s at 94°C, 60 s at 52°C, and 60 s at 72°C, with a final elongation of 10 minutes at 72°C. After amplification, the PCR products were evaluated by electrophoresis in 1.5% agarose gel to ensure amplification had occurred and was the expected size of approximately 400 bp. The PCR product was purified with the Mag-Bind Rxn Pure Plus kit (Omega Bio-Tek, CA, Norcross, GA, USA) by mixing 20 μL of amplicon with 25 μL of Mag-Bind in a 96 well flat-bottom micro-titer plate. After incubation for 5 min at room temperature, the beads were separated and washed twice with 80% ethanol and then eluted in 32 μL of 10 mM Tris pH 8.5 buffer solution. A second PCR was performed to attach dual indices and Illumina® sequencing adapters using the Nextera XT Index kit (Illumina®; San Diego, CA, USA). The conditions of this PCR included: 3 minutes at 94°C followed by 8 cycles of 30 s at 94°C, 30 s at 55°C, and 30 s at 72°C, plus a final step of 10 minutes at 72°C. After purification of these amplicons, the samples were quantified via NanoDrop® spectrophotometry (Thermo Fisher Scientific; Wilmington DE, USA). Normalization and sequencing of the library pool was performed at the Agriculture and Food Laboratory, University of Guelph using an Illumina® MiSeq Reagent kit with a 2 x 250 bp read length. The raw sequence data and the metadata are available in the Scholars Portal Dataverse https://doi.org/10.5683/SP2/7OWFQL.

### Bioinformatics and statistical analyses

Following DNA sequencing of the cytobrush samples, Mothur v1.36.1 was used for sequence processing [19, 20]. Paired end reads were assembled and subjected to a series of quality control steps to remove that were greater than 250 bp in length or with ambiguous base calls or runs of homopolymers greater than 8 bp. Alignment of sequences to the Silva v128 16S rRNA reference database [21] was implemented, with the removal of sequences that did not align with the correct region. VSEARCH v2.0.3 was conducted using the same Silva reference database to identify chimeras [22], which were then removed. Ribosomal Database Project classifier (v14) was used for taxonomic assignment of sequences [23], with subsequent removal of Archaea. Sequences were binned into operational taxonomic units (OTUs) using an open (de novo) picking approach. In order to standardize sequence numbers used for analysis (data normalization), subsampling was completed based on the smallest number of sequences from our dataset [24].

The statistical analyses were performed using RStudio (version 3.6.3; R Core Team, Vienna, Austria) by employing the packages vegan [25], fossil [26], and phyloseq [27]. First, decontamination was done via the decontam R package [28] by using the sequencing from the 6 sterile cytobrush samples exposed to the environment of the barn (in the air for 5 s) in which uterine samples were collected (S1 Fig). For beta diversity (phylum and genera levels), principal coordinate analysis (Bray-Curtis) was used to assess differences in uterine bacterial composition by DIM, using non-parametric multivariate analysis of variance (PERMANOVA). The interaction of DIM at sampling with meloxicam treatment, parity, and serum progesterone category at 35 DIM was evaluated with two-way PERMANOVA with 1000 permutations based on Bray-Curtis dissimilarity with p-values adjusted using the Benjamini-Hochberg correction. Venn diagrams [29] were generated to show the number of bacterial core genera (having ˃ 1% abundance) by DIM at sampling and by meloxicam treatment, parity, and serum progesterone category at 35 DIM. The *simper* function (vegan package in R) was used to set the similarity percentages to extract the most influential bacteria (phylum and genera levels). Alpha diversity indices for bacterial genera (Chao1, Shannon-Weiner, and Camargo’s evenness), and phylum and genera-level data (based on the *simper* function extraction) were analyzed with mixed linear regression models (package lme4) [30]. Alpha diversity data were normally distributed, and relative abundance data varied between 0 and 1 and were not normally distributed (analyzed via Shapiro-Wilk’s test). The relative abundance data were logit-transformed for analysis, with a small bias correction factor for zero values. Linear regression models were fitted for DIM at sampling and their respective interactions with meloxicam treatment, parity, or progesterone category at 35 DIM. Repeated measures were accounted for using an autoregressive type 1 covariance structure in all models and *P*-values were adjusted using the Bonferroni correction. Linear discriminant analysis effect size was performed using an online interface (https://huttenhower.sph.harvard.edu/galaxy/) [31] with meloxicam treatment, parity, or progesterone category at 35 DIM as the main class, DIM at sampling as the subclass, and the cow ID as the subject, setting an alpha of 0.05 and an effect size threshold of 3.5. Heatmaps representing bacteria phyla and genera fold-changes (average linkage clustering based on Bray–Curtis distance) and similarity dendrograms (based on Bray–Curtis distance and unweighted pair group method with arithmetic mean clustering) were built to show bacterial clustering (package Heatplus in R) [32].

## Results

### Descriptive statistics

The data for this non-randomized clinical trial were from 16 healthy Holstein cows that completed the transition period (from calving until 35 DIM) without clinical disease or any antibiotic treatment. The detailed composition of the experimental groups including prepartum BCS, BCS at 35 DIM, average milk production to 35 DIM, average parity per experimental group, and average progesterone concentration at 35 DIM experimental group are shown in S1 Table. None of the cows had PVD at 35 DIM (mucopurulent discharge or worse) and the proportion of endometrial PMN at 10, 21, and 35 DIM per experimental group is summarized in S2 Table.

Sequencing of the V4 region of the bacterial 16S rRNA gene from the 48 endometrial cytobrush samples resulted in 4,945,562 reads, with an average of 103,032 ± 35,382 reads per sample after quality filtering. All effective reads were successfully classified into the phylum and genera levels (˃ 97% sequence identity). Rarefaction curves reached plateau in all samples sequenced, indicating that sampling depth was sufficient to describe uterine microbiota.

### DIM at sampling

**Fig 1:**
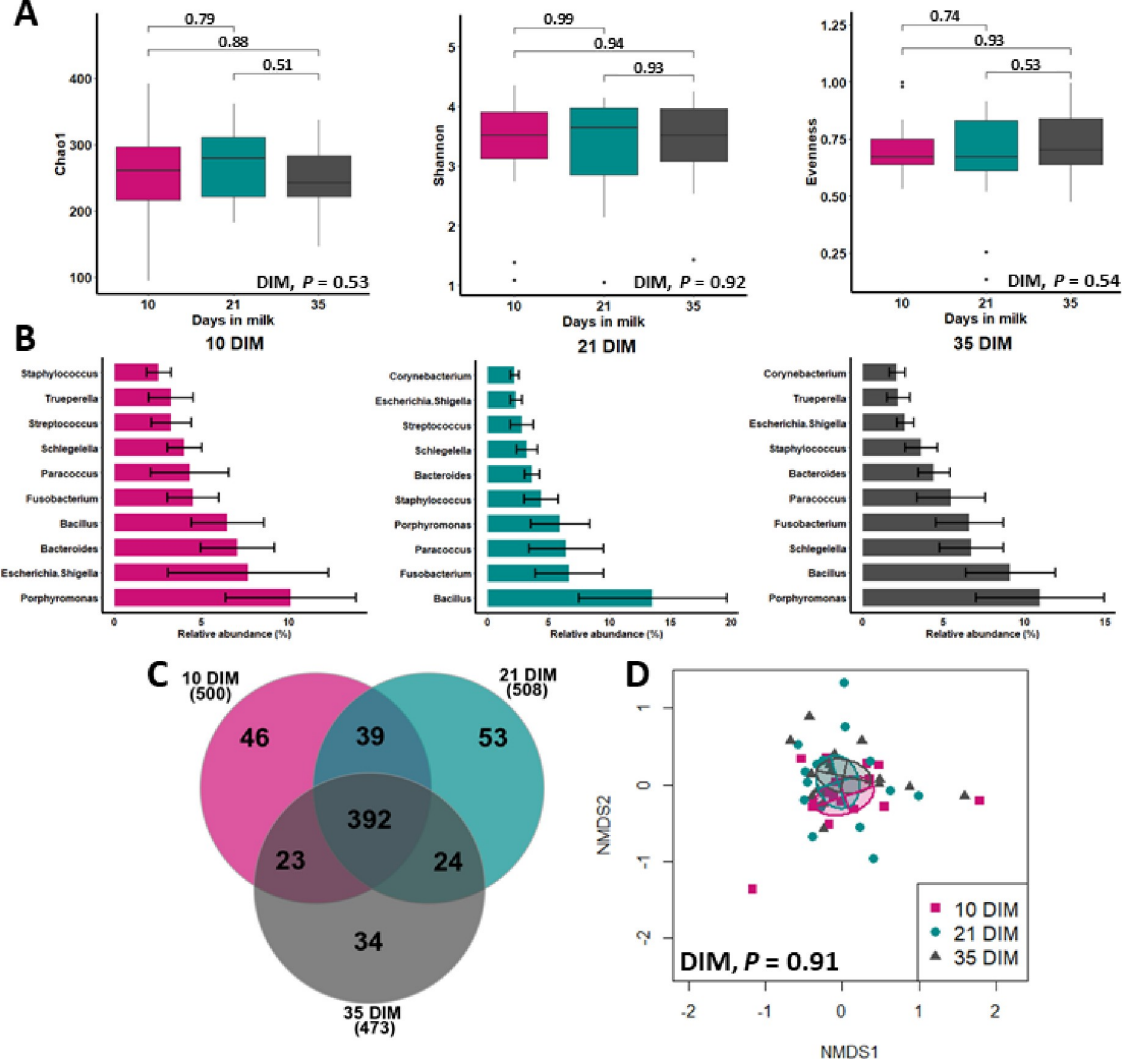
Dynamics of uterine microbiota in clinically healthy postpartum dairy cows (n = 16) in samples collected at 10, 21, and 35 d in milk (DIM). A) Alpha diversity for bacterial genera (numbers above the brackets are *P* values), B) mean relative abundance (with standard error) of the top ten bacteria genera (analyzed via mixed linear regression models), C) number of bacterial core genera (having ˃ 1% abundance), and D) beta diversity (principal coordinate analysis (Bray-Curtis)) were similar among 10, 21, and 35 DIM (analyzed via PERMANOVA with 1000 permutations).

Fig 1A shows no differences in alpha diversity indices (Chao1, Shannon-Weiner, and Camargo’s evenness indices) for bacteria genera at 10, 21, and 35 DIM. The top 10 bacteria relative abundance and the number of bacterial core genera were similar among samples taken at 10, 21, and 35 DIM (Fig 1B and 1C, respectively). The relative abundance of the most influential bacteria phyla was not affected by DIM at sampling (S2 Fig). Principal coordinate analysis based on Bray-Curtis dissimilarity (beta diversity) showed a similar microbiota composition across sampling days at the phyla (S3 Fig) and genera levels (Fig 1D).

### Meloxicam treatment, parity, and blood progesterone

**Fig 2:**
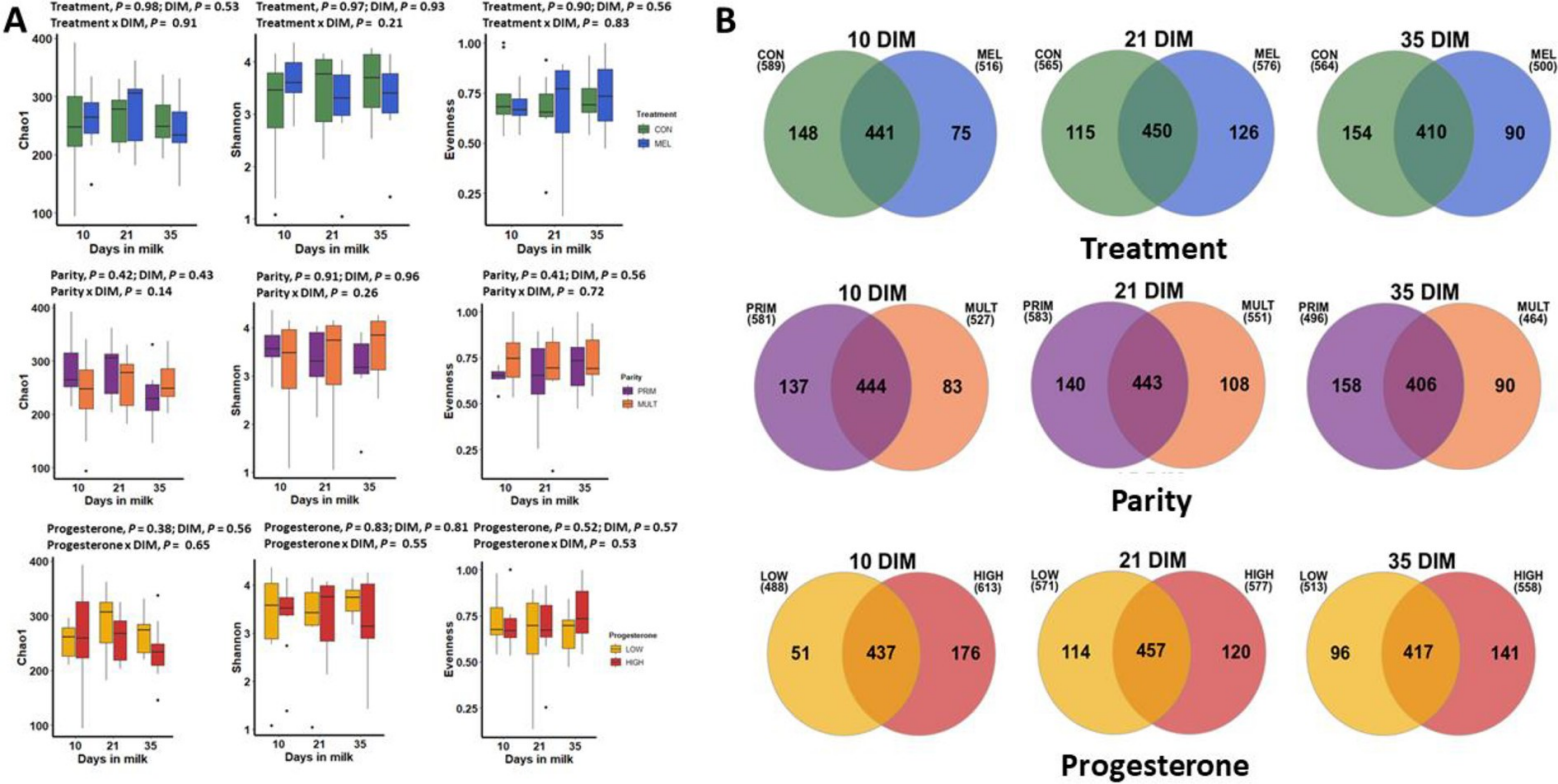
Effects of meloxicam treatment (n = 9, control (CON) and n = 7, meloxicam (MEL)), parity (n = 7, primiparous (PRIM) and n = 9, multiparous (MULT)), and blood progesterone concentration at 35 DIM (n = 10, ˃ 1 ng/mL (HIGH) and n = 6, ≤ 1 ng/mL (LOW)) on the dynamics of the uterine microbiota of clinically healthy postpartum dairy cows (n = 16) in samples collected at 10, 21, and 35 d in milk (DIM). A) Alpha diversity for bacterial genera was not affected by MEL, parity, or progesterone category at 35 DIM (analyzed via mixed linear regression models). B) There number of bacterial core genera (having ˃ 1% abundance) was variable between CON and MEL. PRIM and LOW had more stable, greater numbers of bacterial core genera than MULT and HIGH, respectively.

**Fig 3:**
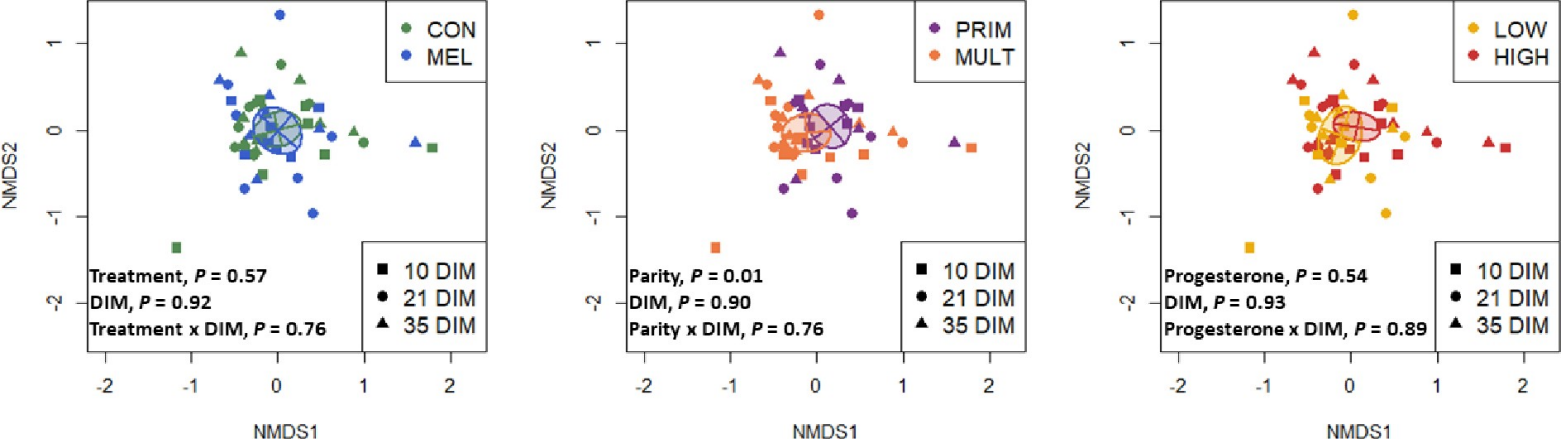
Effects of meloxicam treatment (n = 9, control (CON) and n = 7, meloxicam (MEL)), parity (n = 7, primiparous (PRIM) and n = 9, multiparous (MULT)), and blood progesterone concentration at 35 DIM (n = 10, ˃ 1 ng/mL (HIGH) and n = 6, ≤ 1 ng/mL (LOW)) on the dynamics of the uterine microbiota of clinically healthy postpartum dairy cows (n = 16) in samples collected at 10, 21, and 35 d in milk (DIM). Principal coordinate analysis for Bray-Curtis dissimilarity (genus level) was not affected by MEL or progesterone concentration at 35 DIM. Genera beta diversity (Bray-Curtis dissimilarity) showed differences in microbiome composition between parity groups (analyzed via PERMANOVA with 1000 permutations).

**Fig 4:**
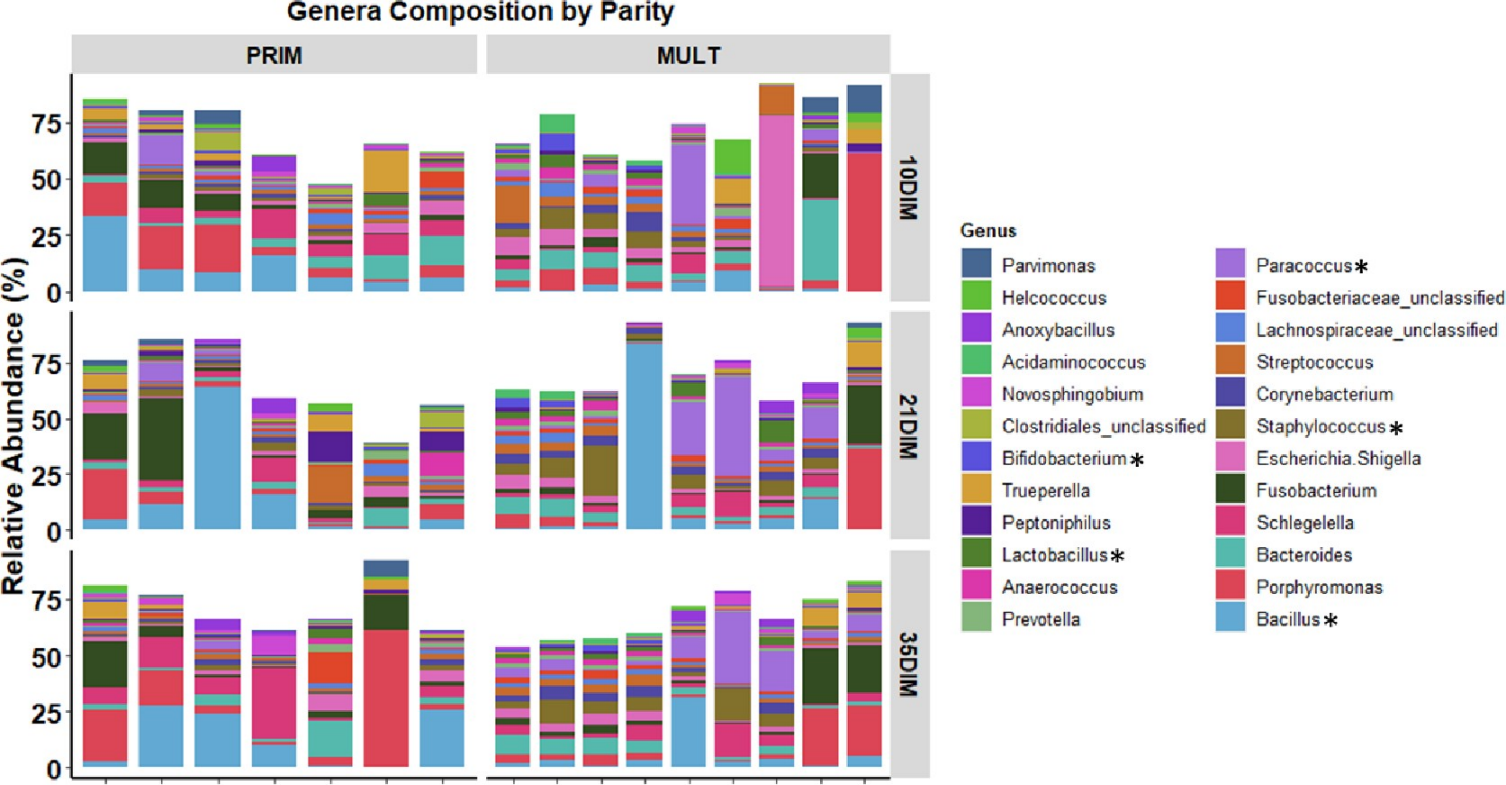
Relative abundance of the most influential bacterial genera in clinically healthy primiparous (PRIM, n = 7) and multiparous (MULT, n = 9) postpartum dairy cows in uterine samples collected at 10, 21, and 35 d in milk (DIM). There was an overall lower abundance of *Anaerococcus*, *Bifidobacterium*, *Corynebacterium*, *Lactobacillus*, *Paracoccus*, *Staphylococcus*, and *Streptococcus* and higher abundance of *Bacillus*, *Fusobacterium*, and *Novosphingobium* in PRIM than MULT (*P* < 0.05). Asterisks indicate differences between parity groups analyzed via mixed linear regression models.

**Fig 5:**
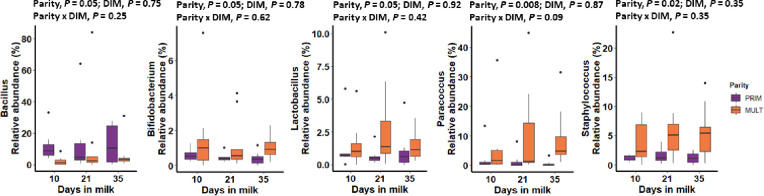
Relative abundance (by days in milk (DIM)) of bacterial genera that differed between clinically healthy primiparous (PRIM, n = 7) and multiparous (MULT, n = 9) postpartum dairy cows in uterine samples collected at 10, 21, and 35 DIM. Relative abundance data were analyzed via mixed linear regression models accounting for repeated measures.

**Fig 6:**
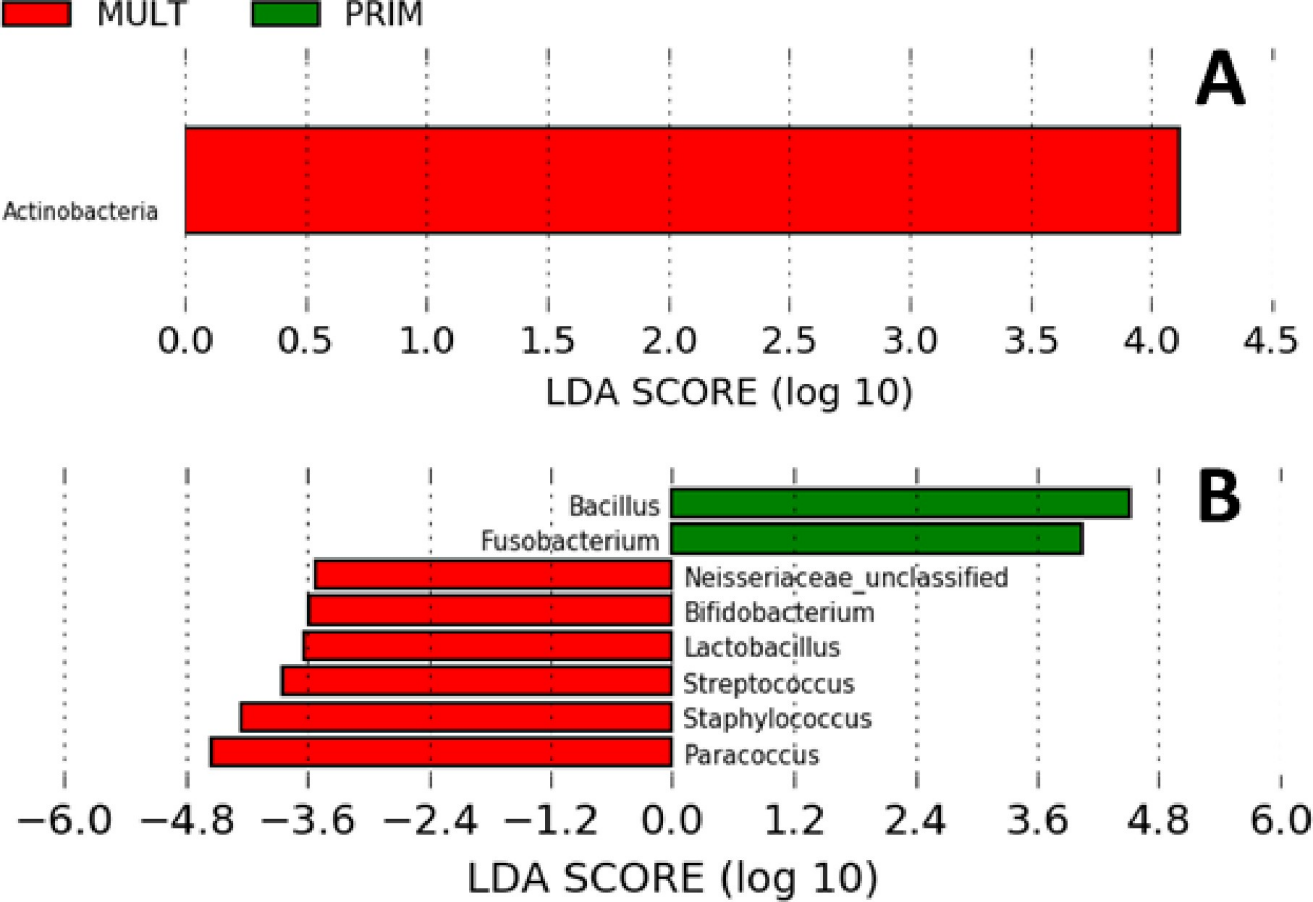
Linear discriminant analysis (LDA) effect size plots showing the differences in uterine microbiota between primiparous (PRIM; n = 7) and multiparous (MULT; n = 9) cows in samples collected at 10, 21, and 35 d in milk (DIM). Histograms show the LDA effect size computed for features at the phyla (A) and genera (B) levels. Enriched features for PRIM are indicated with positive LDA scores (green), and enriched features in MULT cows are indicated with negative LDA scores (red). Only features with *P* ˃ 0.05 and an effect size cut-off of 3.5 are plotted.

**Fig 7:**
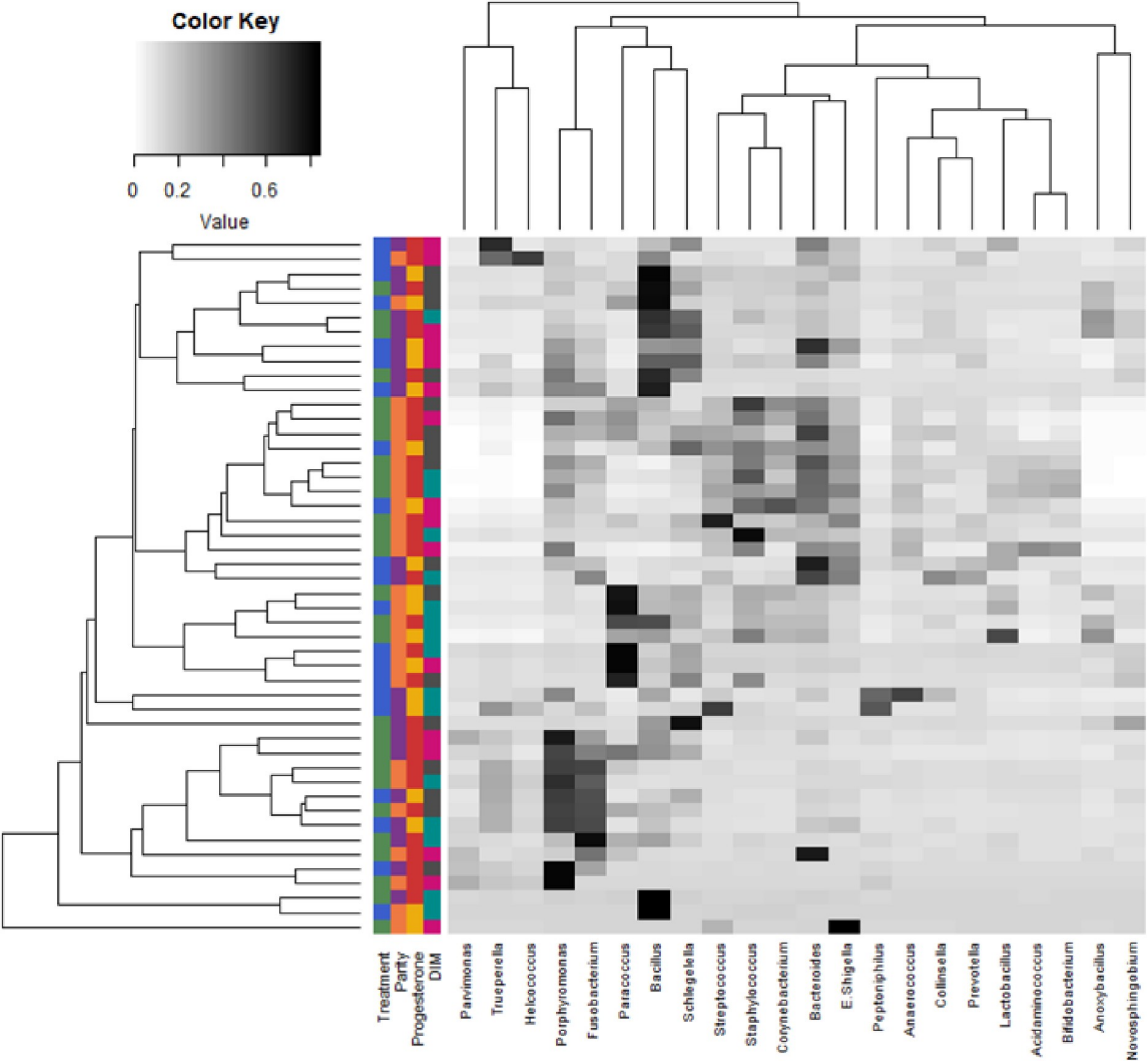
Heatmap with average linkage clustering based on Bray–Curtis distance showing the relative abundances of the most influential uterine bacteria genera in clinically healthy postpartum dairy cows (n = 16) in samples collected at 10, 21, and 35 d in milk (DIM). The relative abundance is indicated by a gradient of color from black (high abundance) to white (low abundance). Similarity dendrogram shows the unweighted pair group method with arithmetic mean (UPGMA) clustering of bacterial genera by treatment (n = 9, control (green) and n = 7, meloxicam (blue)), parity (n = 7, primiparous (purple) and n = 9, multiparous (orange), blood progesterone concentration at 35 DIM (n = 10, ˃ 1 ng/mL (red) and n = 6, ≤ 1 ng/mL (yellow)), and DIM (10 DIM (pink), 21 DIM (turquoise), and 35 DIM (dark grey)).

Alpha diversity for bacterial genera was not associated with meloxicam treatment, parity, or progesterone category at 35 DIM (Fig 2A). The core genera (Venn diagram) stratified by meloxicam treatment, parity, and progesterone category at 35 DIM are illustrated in Fig 2B. Principal coordinate analysis for Bray-Curtis dissimilarity (genus level) was not affected by meloxicam treatment or progesterone category at 35 DIM (Fig 3). Beta diversity (Bray-Curtis dissimilarity) at the genus level showed differences in microbiota composition between parity groups (Fig 3). Beta diversity at the phyla level was not affected by meloxicam treatment, parity, or progesterone category at 35 DIM (S4 Fig). The relative abundance of the most influential bacteria phyla and genera are shown in Fig 4 and in S5–S9 Figs. There was lesser relative abundance of *Bifidobacterium*, *Lactobacillus*, *Paracoccus*, and *Staphylococcus* and greater abundance of *Bacillus* in primiparous than multiparous cows (Fig 5) as indicated by the linear regression models. LDA-LEfSe showed a greater discriminant score of Actinobacteria in multiparous than primiparous cows (phylum level; Fig 6A). At the genera level, primiparous cows had discriminately greater *Bacillus* and *Fusobacterium* and discriminately lesser *Neisseriaceae*, *Bifidobacterium*, *Lactobacillus*, *Streptococcus*, *Staphylococcus*, and *Paracoccus* than multiparous cows (Fig 6B). Linear regression and LDA-LEfSe did not find an effect of meloxicam treatment or progesterone category on the microbiota composition at the phylum or genus levels. The heatmap and similarity dendrograms in Fig 7 show a more similar clustering for bacterial genera in parity groups than for meloxicam or progesterone category at 35 DIM. At the phyla level (S10 Fig), heatmap and similarity dendrograms show similar tendencies as for the genera level (more similar clustering in parity groups).

## Discussion

This study primarily focused on describing the effects of NSAID treatment and other factors that may affect the dynamics of bacterial populations by using 16S rRNA sequencing of endometrial samples obtained from the postpartum uterus of clinically healthy dairy cows. Neither meloxicam treatment (from 10 to 13 DIM) nor DIM at sampling (10, 21, and 35) influenced the structure or composition of the microbiota found in the postpartum uterus. Beta diversity (Bray-Curtis dissimilarity) at the genus level showed differences in microbiota composition between parity groups, with differences in the relative abundance of several bacterial genera. Progesterone category at 35 DIM did not have an effect on the uterine microbiota.

We aimed to investigate the uterine bacteria dynamics in clinically healthy dairy cows after the vaginal-uterine decompartmentalization process. In this regard, Miranda-CasoLuengo et al. [11] demonstrated that compartmentalization between the vagina and the uterine microbiota is re-established after 7 DIM in clinically healthy cows. Therefore, we decided to analyze the microbiota of our uterine samples starting at 10 DIM. Moreover, sampling the endometrium within the first week postpartum can be challenging. Based on our experience, uterine sampling within the first week postpartum can be difficult because in early stages of involution many cows have large, pendulous uteri that cannot be properly catheterized. In humans, transcervical uterine sampling is described as a cause of cross-contamination with the cervical microbiota [33].

Certain generalizations can be made from the existing literature, but there is no consensus on the nature of a “healthy” uterine microbiota [33]. Although none of the cows in our study had clinical disease, there was variation in the proportions of endometrial PMN. Wang et al. [5] and Pascottini et al. [6] showed that neither the alpha and beta diversities nor the relative abundance of bacteria phyla and genera differed between healthy and SCE cows. However, it was shown that in cows with PVD (or clinical endometritis), there was an increased relative abundance of *Trueperella* in comparison to healthy or SCE [5, 6]. None of the cows in the present study had PVD (mucopurulent vaginal discharge or worse) at 35 DIM. Therefore, we consider that our results are not confounded by the presence of SCE at 35 DIM in some of the cows in this experiment.

Our results clearly show that a uterine microbiota was similar at 10, 21, 35 DIM. At each time point, Firmicutes was the most abundant phylum followed by Proteobacteria, Bacteroidetes, and Actinobacteria. At the genus level, *Bacillus*, *Fusobacterium*, and *Porphyromonas* were (interchangeably) within top 5 bacteria with the greatest relative abundance. However, the sum of their relative abundances was less than 30% of the total bacteria population. This highlights the tremendous bacterial diversity encountered by the uterus of clinically healthy postpartum cows. Increased abundance of *Bacteroides*, *Porphyromonas*, and *Fusobacterium* accompanied with loss of heterogeneity was found in cows that later developed metritis [9, 34]. The total relative abundance of these bacterial genera was over 60% of the uterine microbiota of metritic cows [10, 34]. Potential pathogenic bacteria are present in the uterus of postpartum cows; however, high microbial diversity apparently is the key to a healthy uterine environment [9, 33].

Traditionally, the therapeutic use of NSAID is effective at blocking the conversion of arachidonic acid to prostaglandins by inhibiting the inducible cyclooxygenase-2 pathway [35]. Moreover, it was also demonstrated that NSAID could down-regulate the inflammatory response by inhibiting pro-inflammatory cytokine production, as well as suppression of nuclear factor-kappa B activity [36]. Therefore, our hypothesis was that if we reduce systemic and uterine inflammation, this would potentially affect uterine bacterial composition. However, meloxicam treatment did not affect the endometrial inflammatory status [14]. Consequently, here we show that meloxicam affected neither the alpha or beta microbiota diversities nor relative abundances of bacteria at the phylum or genus levels. Nevertheless, it is important to mention that our meloxicam treatment was from 10 to 13 DIM, so from the last meloxicam injection to the next endometrial sample for 16S rRNA sequencing there was a lag of 7 days. Our rationale for analyzing samples collected at 21 DIM was based on an expected delay of meloxicam action on the uterine microbiota that would be detectable one week after the last meloxicam treatment. Thus, this study demonstrated the lack of effect of meloxicam on uterine microbiota in the medium term. The short-term effect of NSAIDs on the uterine microbiota remains uncertain.

Our principal coordinate analysis (beta diversity) for bacteria genera demonstrates that the uterine microbiotas from primiparous and multiparous differ from each other. The difference between parities might be attributable to the core resident bacteria in cows that have previously calved. Also, differences in anatomy and involution between older cows and those just calved for the first time may affect the vaginal-uterine bacterial exchange. Furthermore, the differences in the microbiota associated with parity might be also attributable to changes occurring at parturition. Bicalho et al. [37] found greater diversity but with lower total bacterial counts in vaginal samples collected just after calving in multiparous than primiparous cows. The increased likelihood of bacterial contamination in primiparous cows could be related to a longer or more difficult parturition (although eutocic in the present data), resulting in increased trauma or altered neutrophil function [38, 39]. On the other hand, differences in metabolism and the characteristic greater milk production in multiparous could also contribute to differences in their uterine bacterial composition. Meloxicam induced changes in the metabolic profile by decreasing haptoglobin and improving markers of energy metabolism [14], but without effects on the uterine microbiota. However, these metabolic changes were transient and returned to basal levels just after the end of anti-inflammatory treatment. Metabolic adaptations associated with milk production associated with multiparity are more profound and persistent, and it had shown to play a vital role in the development of postpartum uterine disease [40].

This study shows that progesterone serum concentration at 35 DIM was not associated with distinct uterine microbiota. We hypothesized that the endometrial changes associated with increased circulating progesterone concentration (indicating that ovulation had occurred) could affect the uterine microbiota [12]. Because few cows are expected to have a functional corpus luteum by 10 or 21 DIM, we did not measure the blood progesterone concentrations at these time points. Our data do not refute possible effects of progesterone on the uterine microbiota later in lactation in estrus cyclic cows.

## Conclusions

The uterine microbiota in endometrial cytobrush samples collected from clinically healthy postpartum dairy cows was not different at 10, 21, or 35 DIM. Meloxicam treatment from 10 to 13 DIM did not affect the uterine microbiota. However, there could have been transient microbiota changes (during or just after meloxicam treatment) that in this study were not assessed. There was greater diversity in primiparous than multiparous cows, with greater relative abundance in bacterial genera *Bacillus* and *Fusobacterium* and lesser relative abundance of *Anaerococcus*, *Bifidobacterium*, *Lactobacillus*, *Neisseriaceae*, *Paracoccus*, *Staphylococcus*, and *Streptococcus*. Progesterone serum category at 35 DIM was not associated with the uterine bacterial composition. Nevertheless, this study has some limitations associated with the (relatively) small sample size and lack of randomization in selecting the cows. We encourage further characterization of the establishment of the uterine microbiota in young cattle, and how it changes with the first and subsequent calvings. Further studies should be performed to evaluate the effects of sex hormones (estrogens and progesterone) systemically and locally on the uterine microbiota of dairy cows.

## Supporting information

S1 Fig Relative abundance of bacterial phyla and genera of six sterile cytobrush samples exposed to the air of the barn where the experimental cows were sampled (clean cytobrush samples). These samples were used for decontamination of the data.
(DOCX)

S2 Fig Average relative abundances of the most influential bacterial phyla in clinically healthy postpartum dairy cows (n = 16) in samples collected at 10, 21, and 35 d in milk (DIM). Uterine bacteria phyla did not differ by DIM (*P* ˃ 0.3), analyzed via mixed linear regression models.
(DOCX)

S3 Fig Dynamics of uterine microbiota in clinically healthy postpartum dairy cows (n = 16) in samples collected at 10, 21, and 35 d in milk (DIM). Beta diversity (principal coordinate analysis (Bray-Curtis)) for bacteria phyla were similar at 10, 21, and 35 DIM (analyzed via PERMANOVA with 1000 permutations).
(DOCX)

S4 Fig Effects of meloxicam treatment (n = 9, control (CON) and n = 7, meloxicam (MEL)), parity (n = 9, primiparous (PRIM) and n = 7, multiparous (MULT)), and blood progesterone concentration at 35 DIM (n = 10, ˃ 1 ng/mL (HIGH) and n = 6, ≤ 1 ng/mL (LOW)) on the dynamics of uterine microbiota of clinically healthy postpartum dairy cows (n = 16) in samples collected at 10, 21, and 35 d in milk (DIM). Principal coordinate analysis for Bray-Curtis dissimilarity (phylum level) was not affected by MEL, parity groups, or blood progesterone concentration at 35 DIM (analyzed via PERMANOVA with 1000 permutations).
(DOCX)

S5 Fig Relative abundance of the most influential bacterial phyla in clinically healthy postpartum dairy cows in samples collected at 10, 21, and 35 d in milk (DIM). Cows received meloxicam (MEL, 0.5 mg/kg SC, n = 7) once daily for 4 d (10–13 d in milk (DIM)) or were untreated (CON, n = 9). MEL did not affect the uterine bacteria phyla relative abundance (*P* ˃ 0.2; analyzed via mixed linear regression models).
(DOCX)

S6 Fig Relative abundance of the most influential bacterial phyla in clinically healthy primiparous (PRIM, n = 7) and multiparous (MULT, n = 9) postpartum dairy cows in samples collected at 10, 21, and 35 d in milk (DIM). MULT had greater abundance of *Actinobacteria* than PRIM (*P* = 0.02; analyzed via mixed linear regression models).
(DOCX)

S7 Fig Relative abundance of the most influential bacterial phyla in clinically healthy postpartum dairy cows in samples collected at 10, 21, and 35 d in milk (DIM). Progesterone concentration in blood samples at 35 DIM were classified as ˃ 1 ng/mL (HIGH, n = 10) or ≤ 1 ng/mL (LOW, n = 6). Progesterone concentration at 35 DIM did not affect the uterine bacteria phyla relative abundance (*P* ˃ 0.2; analyzed via mixed linear regression models).
(DOCX)

S8 Fig Relative abundance of the most influential bacterial genera in healthy postpartum dairy cows in samples collected at 10, 21, and 35 d in milk (DIM). Cows received meloxicam treatment (MEL, 0.5 mg/kg SC, n = 7) once daily for 4 d (10–13 d in milk (DIM)) or were untreated (CON, n = 9). MEL did not affect the uterine bacteria genera relative abundance (*P* ˃ 0.1; analyzed via mixed linear regression models).
(DOCX)

S9 Fig Relative abundance of the most influential bacterial genera in clinically healthy postpartum dairy cows in samples collected at 10, 21, and 35 d in milk (DIM). Progesterone concentration in blood samples at 35 DIM were classified as ˃ 1 ng/mL (HIGH, n = 10) or ≤ 1 ng/mL (LOW, n = 6). Progesterone concentration at 35 DIM did not affect the uterine bacterial genera relative abundance (*P* ˃ 0.1; analyzed via mixed linear regression models).
(DOCX)

S10 Fig Heatmap with average linkage clustering based on Bray–Curtis distance showing the relative abundances of the most influential uterine bacteria phyla in clinically healthy postpartum dairy cows (n = 16) in samples collected at 10, 21, and 35 d in milk (DIM). The relative abundance is indicated by a gradient of color from black (high abundance) to white (low abundance). Similarity dendrogram shows the unweighted pair group method with arithmetic mean (UPGMA) clustering of bacterial genera by treatment (n = 9, control (green) and n = 7, meloxicam (blue)), parity (n = 7, primiparous (purple) and n = 9, multiparous (orange), blood progesterone concentration at 35 DIM (n = 10, ˃ 1 ng/mL (red) and n = 6, ≤ 1 ng/mL (yellow)), and DIM (10 DIM (pink), 21 DIM (turquoise), and 35 DIM (dark grey)).
(DOCX)

S1 Table Composition of groups of clinically healthy postpartum Holstein cows from which endometrial cytobrush samples were collected at 10, 21, and 35 d in milk (DIM) to study their uterine microbiome. (DOCX)

S2 Table Endometrial polymorphonuclear proportions (PMN%) in clinically healthy postpartum Holstein cows from which endometrial cytobrush samples were collected at 10, 21, and 35 d in milk (DIM) to study their uterine microbiome. (DOCX)
