# Supplementary material for: Effects of parity, blood progesterone, and non-steroidal anti-inflammatory treatment on the dynamics of the uterine microbiota of healthy postpartum dairy cows
Source: PLoS One. 2021 Feb 19;16(2):e0233943. doi: 10.1371/journal.pone.0233943 (PMC7895344; doi:10.1371/journal.pone.0233943)
Supplement: S1 Fig — These samples were used for decontamination of the data. (DOCX) [file pone.0233943.s001.docx]

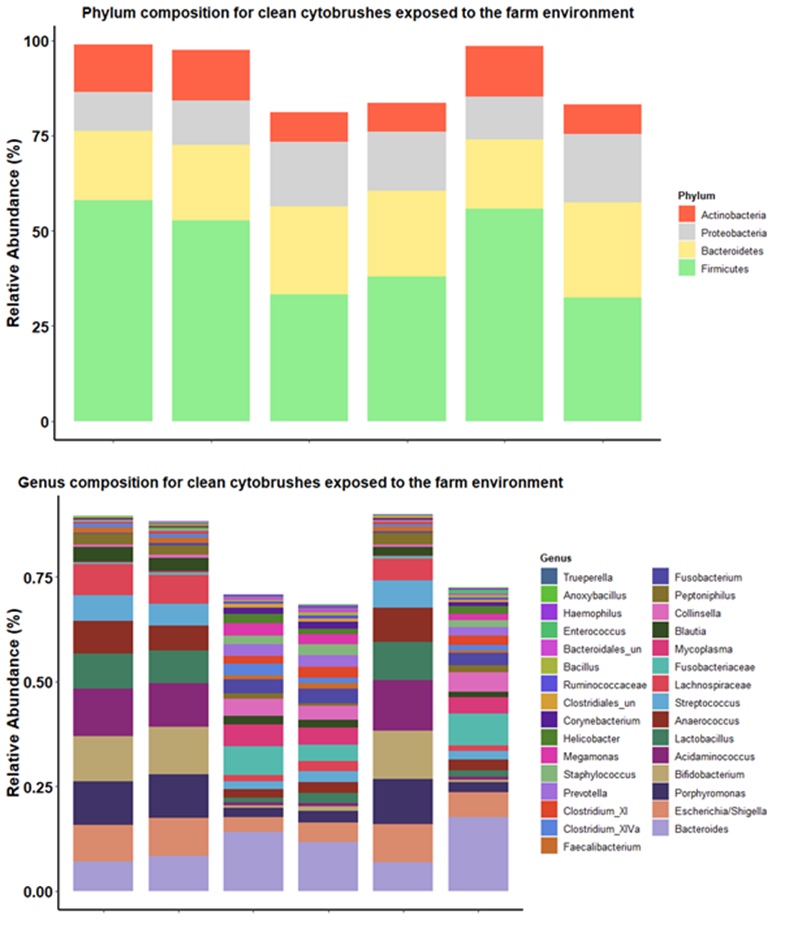


**S1 Fig.** Relative abundance of bacterial phyla and genera of six sterile cytobrush samples exposed to the air of the barn where the experimental cows were sampled (clean cytobrush samples). These samples were used for decontamination of the data.
