# Supplementary material for: Effects of parity, blood progesterone, and non-steroidal anti-inflammatory treatment on the dynamics of the uterine microbiota of healthy postpartum dairy cows
Source: PLoS One. 2021 Feb 19;16(2):e0233943. doi: 10.1371/journal.pone.0233943 (PMC7895344; doi:10.1371/journal.pone.0233943)
Supplement: S2 Fig — Uterine bacteria phyla did not differ by DIM (P ˃ 0.3), analyzed via mixed linear regression models. (DOCX) [file pone.0233943.s002.docx]

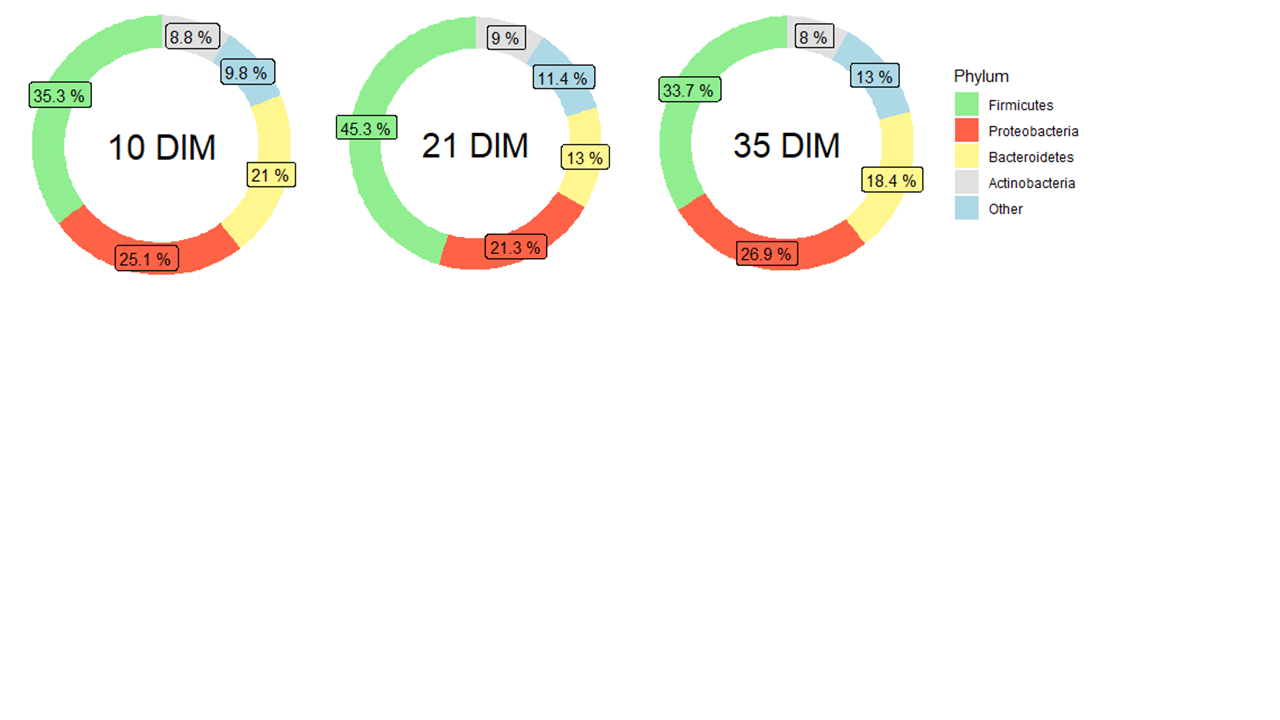


**S2 Fig.** Average relative abundances of the most influential bacterial phyla in clinically healthy postpartum dairy cows (n = 16) in samples collected at 10, 21, and 35 d in milk (DIM). Uterine bacteria phyla did not differ by DIM (*P* ˃ 0.3), analyzed via mixed linear regression models.
