# Supplementary material for: Effects of parity, blood progesterone, and non-steroidal anti-inflammatory treatment on the dynamics of the uterine microbiota of healthy postpartum dairy cows
Source: PLoS One. 2021 Feb 19;16(2):e0233943. doi: 10.1371/journal.pone.0233943 (PMC7895344; doi:10.1371/journal.pone.0233943)
Supplement: S3 Fig — Beta diversity (principal coordinate analysis (Bray-Curtis)) for bacteria phyla were similar at 10, 21, and 35 DIM (analyzed via PERMANOVA with 1000 permutations). (DOCX) [file pone.0233943.s003.docx]

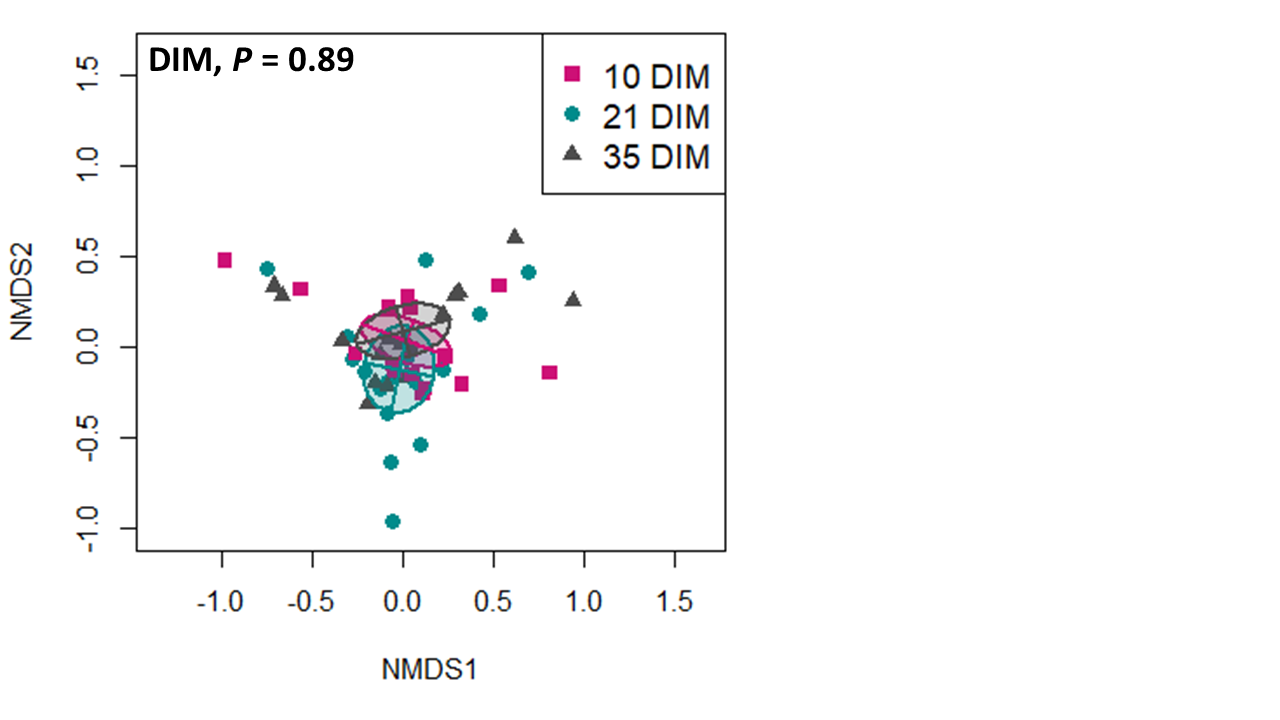


**S3 Fig.** Dynamics of uterine microbiota in clinically healthy postpartum dairy cows (n = 16) in samples collected at 10, 21, and 35 d in milk (DIM). Beta diversity (principal coordinate analysis (Bray-Curtis)) for bacteria phyla were similar at 10, 21, and 35 DIM (analyzed via PERMANOVA with 1000 permutations).
