# Supplementary material for: Effects of parity, blood progesterone, and non-steroidal anti-inflammatory treatment on the dynamics of the uterine microbiota of healthy postpartum dairy cows
Source: PLoS One. 2021 Feb 19;16(2):e0233943. doi: 10.1371/journal.pone.0233943 (PMC7895344; doi:10.1371/journal.pone.0233943)
Supplement: S4 Fig — Principal coordinate analysis for Bray-Curtis dissimilarity (phylum level) was not affected by MEL, parity groups, or blood progesterone concentration at 35 DIM (analyzed via PERMANOVA with 1000 permutations). (DOCX) [file pone.0233943.s004.docx]

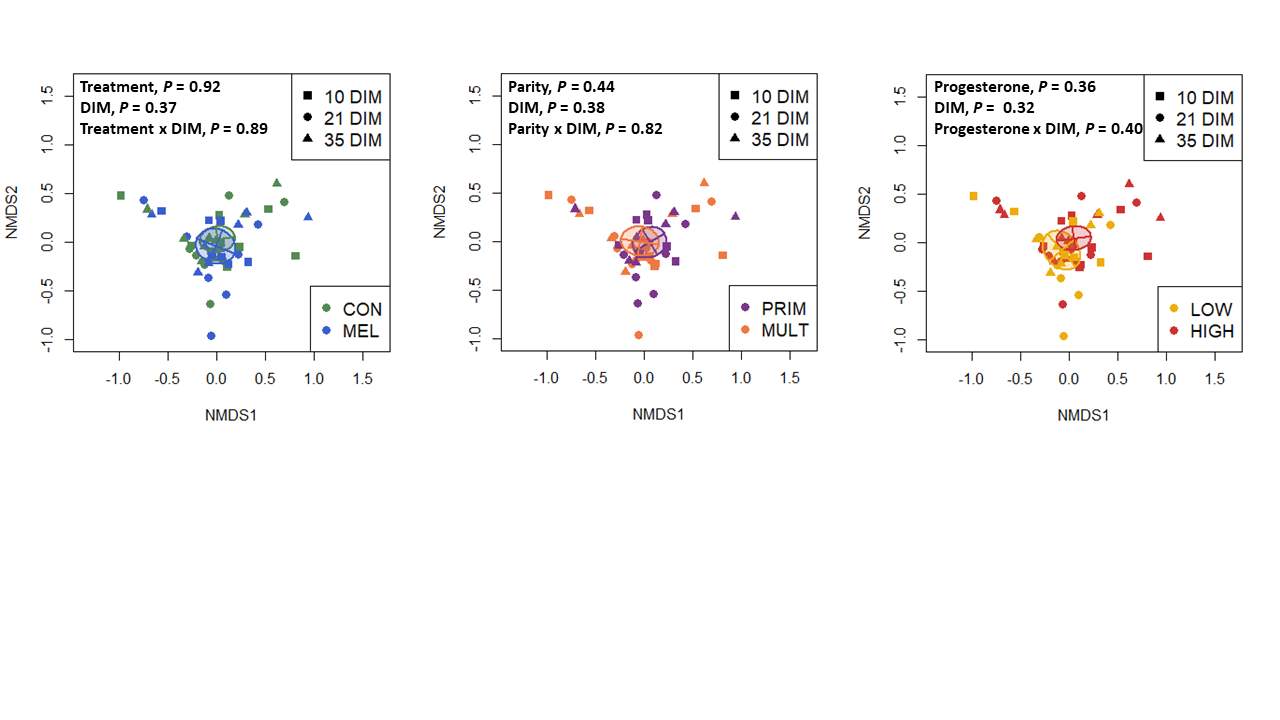


**S4 Fig.** Effects of meloxicam treatment (n = 9, control (CON) and n = 7, meloxicam (MEL)), parity (n = 9, primiparous (PRIM) and n = 7, multiparous (MULT)), and blood progesterone concentration at 35 DIM (n = 10, ˃ 1 ng/mL (HIGH) and n = 6, ≤ 1 ng/mL (LOW)) on the dynamics of uterine microbiota of clinically healthy postpartum dairy cows (n = 16) in samples collected at 10, 21, and 35 d in milk (DIM). Principal coordinate analysis for Bray-Curtis dissimilarity (phylum level) was not affected by MEL, parity groups, or blood progesterone concentration at 35 DIM (analyzed via PERMANOVA with 1000 permutations).
