# Supplementary material for: Effects of parity, blood progesterone, and non-steroidal anti-inflammatory treatment on the dynamics of the uterine microbiota of healthy postpartum dairy cows
Source: PLoS One. 2021 Feb 19;16(2):e0233943. doi: 10.1371/journal.pone.0233943 (PMC7895344; doi:10.1371/journal.pone.0233943)
Supplement: S6 Fig — MULT had greater abundance of Actinobacteria than PRIM (P = 0.02; analyzed via mixed linear regression models). (DOCX) [file pone.0233943.s006.docx]

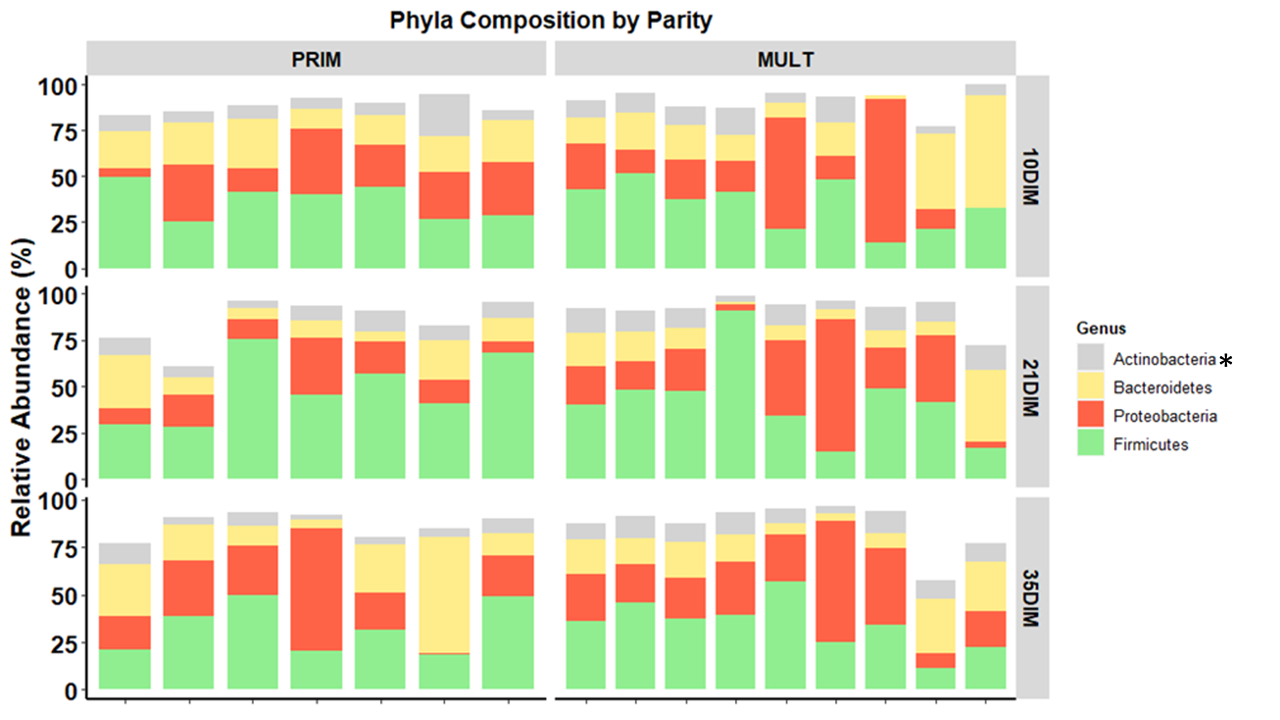


**S6 Fig.** Relative abundance of the most influential bacterial phyla in clinically healthy primiparous (PRIM, n = 7) and multiparous (MULT, n = 9) postpartum dairy cows in samples collected at 10, 21, and 35 d in milk (DIM). MULT had greater abundance of *Actinobacteria* than PRIM (*P* = 0.02; analyzed via mixed linear regression models).
