# Supplementary material for: Effects of parity, blood progesterone, and non-steroidal anti-inflammatory treatment on the dynamics of the uterine microbiota of healthy postpartum dairy cows
Source: PLoS One. 2021 Feb 19;16(2):e0233943. doi: 10.1371/journal.pone.0233943 (PMC7895344; doi:10.1371/journal.pone.0233943)
Supplement: S7 Fig — Progesterone concentration in blood samples at 35 DIM were classified as ˃ 1 ng/mL (HIGH, n = 10) or ≤ 1 ng/mL (LOW, n = 6). Progesterone concentration at 35 DIM did not affect the uterine bacteria phyla relative abundance (P ˃ 0.2; analyzed via mixed linear regression models). (DOCX) [file pone.0233943.s007.docx]

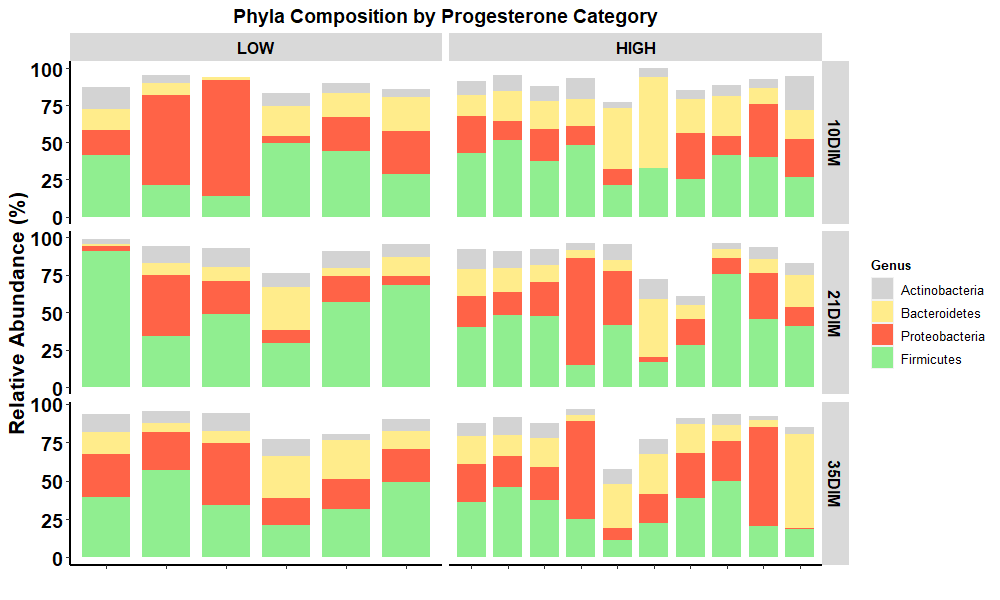


**S7 Fig.** Relative abundance of the most influential bacterial phyla in clinically healthy postpartum dairy cows in samples collected at 10, 21, and 35 d in milk (DIM). Progesterone concentration in blood samples at 35 DIM were classified as ˃ 1 ng/mL (HIGH, n = 10) or ≤ 1 ng/mL (LOW, n = 6). Progesterone concentration at 35 DIM did not affect the uterine bacteria phyla relative abundance (*P* ˃ 0.2; analyzed via mixed linear regression models).
