# Supplementary material for: Effects of parity, blood progesterone, and non-steroidal anti-inflammatory treatment on the dynamics of the uterine microbiota of healthy postpartum dairy cows
Source: PLoS One. 2021 Feb 19;16(2):e0233943. doi: 10.1371/journal.pone.0233943 (PMC7895344; doi:10.1371/journal.pone.0233943)
Supplement: S8 Fig — Cows received meloxicam treatment (MEL, 0.5 mg/kg SC, n = 7) once daily for 4 d (10–13 d in milk (DIM)) or were untreated (CON, n = 9). MEL did not affect the uterine bacteria genera relative abundance (P ˃ 0.1; analyzed via mixed linear regression models). (DOCX) [file pone.0233943.s008.docx]

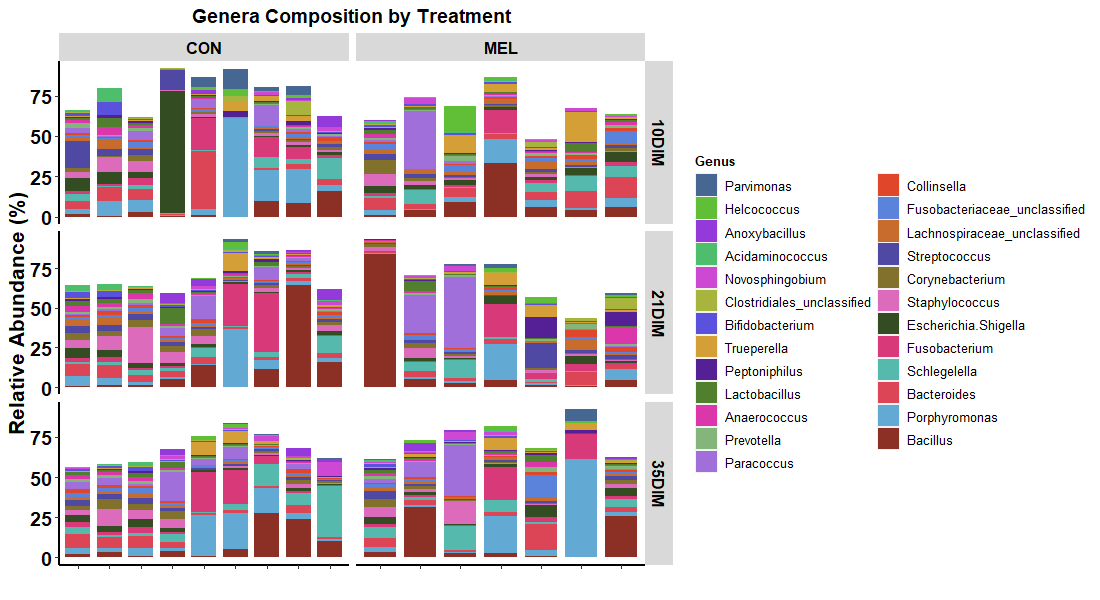


**S8 Fig.** Relative abundance of the most influential bacterial genera in healthy postpartum dairy cows in samples collected at 10, 21, and 35 d in milk (DIM). Cows received meloxicam treatment (MEL, 0.5 mg/kg SC, n = 7) once daily for 4 d (10-13 d in milk (DIM)) or were untreated (CON, n = 9). MEL did not affect the uterine bacteria genera relative abundance (*P* ˃ 0.1; analyzed via mixed linear regression models).
