# Supplementary material for: Effects of parity, blood progesterone, and non-steroidal anti-inflammatory treatment on the dynamics of the uterine microbiota of healthy postpartum dairy cows
Source: PLoS One. 2021 Feb 19;16(2):e0233943. doi: 10.1371/journal.pone.0233943 (PMC7895344; doi:10.1371/journal.pone.0233943)
Supplement: S10 Fig — The relative abundance is indicated by a gradient of color from black (high abundance) to white (low abundance). Similarity dendrogram shows the unweighted pair group method with arithmetic mean (UPGMA) clustering of bacterial genera by treatment (n = 9, control (green) and n = 7, meloxicam (blue)), parity (n = 7, primiparous (purple) and n = 9, multiparous (orange), blood progesterone concentration at 35 DIM (n = 10, ˃ 1 ng/mL (red) and n = 6, ≤ 1 ng/mL (yellow)), and DIM (10 DIM (pink), 21 DIM (turquoise), and 35 DIM (dark grey)). (DOCX) [file pone.0233943.s010.docx]

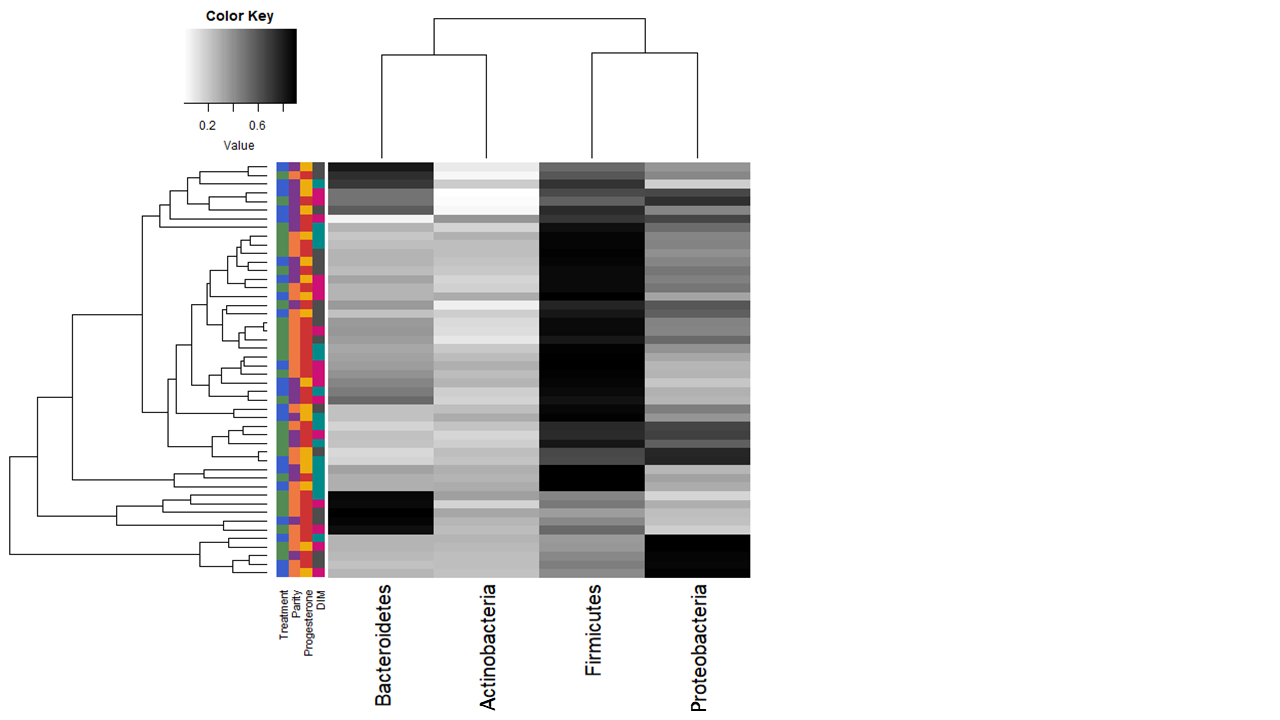


**S10 Fig.** Heatmap with average linkage clustering based on Bray–Curtis distance showing the relative abundances of the most influential uterine bacteria phyla in clinically healthy postpartum dairy cows (n = 16) in samples collected at 10, 21, and 35 d in milk (DIM). The relative abundance is indicated by a gradient of color from black (high abundance) to white (low abundance). Similarity dendrogram shows the unweighted pair group method with arithmetic mean (UPGMA) clustering of bacterial genera by treatment (n = 9, control (green) and n = 7, meloxicam (blue)), parity (n = 7, primiparous (purple) and n = 9, multiparous (orange), blood progesterone concentration at 35 DIM (n = 10, ˃ 1 ng/mL (red) and n = 6, ≤ 1 ng/mL (yellow)), and DIM (10 DIM (pink), 21 DIM (turquoise), and 35 DIM (dark grey)).
