# Supplementary material for: Effects of parity, blood progesterone, and non-steroidal anti-inflammatory treatment on the dynamics of the uterine microbiota of healthy postpartum dairy cows
Source: PLoS One. 2021 Feb 19;16(2):e0233943. doi: 10.1371/journal.pone.0233943 (PMC7895344; doi:10.1371/journal.pone.0233943)
Supplement: S1 Table — (DOCX) [file pone.0233943.s011.docx]

| **S1 Table.** Composition of groups of clinically healthy postpartum Holstein cows from which endometrial cytobrush samples were collected at 10, 21, and 35 d in milk (DIM) to study their uterine microbiome. | | | | | | |
| --- | --- | --- | --- | --- | --- | --- |
| **Group^1^** | | **BCS pre-partum** | **BCS 35 DIM** | **Average milk production** | **Parity** | **Progesterone (ng/mL)** |
| **Treatment** | **CON** (n = 9) | 3.7 ± 0.1 | 2.9 ± 0.2 | 32.2 ± 8.0 | 2.1 ± 1.8 | 1.2 ± 2.2 |
|  | **MEL** (n = 7) | 3.8 ± 0.1 | 3.1 ± 0.1 | 33.3 ± 9.2 | 1.4 ± 0.4 | 2 ± 1.7 |
| **Parity** | **PRIM** (n = 7) | 3.7 ± 1.8 | 3.0 ± 0.1 | 24.7 ± 3.3 | 1 ± 0 | 2.8 ± 2.4 |
|  | **MULT** (n = 9) | 3.8 ± 2 | 3.0 ± 0.2 | 37.7 ± 6.4 | 2.3 ± 1.7 | 2.6 ± 2.7 |
| **Progesterone** | **LOW** (n = 6) | 3.8 ± 0.1 | 2.9 ± 0.2 | 34.6 ± 7.3 | 1.2 ± 0.5 | 0.1 ± 0.1 |
|  | **HIGH** (n = 10) | 3.7 ± 0.2 | 3.0 ± 0.1 | 31.6 ± 9.1 | 2 ± 1.7 | 4.3 ± 1.6 |
| ^1^CON, control cows; MEL, cows received meloxicam (MEL, 0.5 mg/kg SC, n = 11) once daily for 4 d (10-13 (DIM)); PRIM, primiparous cows; MULT; multiparous cows; LOW, ≤ 1 ng/mL blood progesterone concentration at 35 DIM ;HIGH, ˃ 1 ng/mL blood progesterone concentration at 35 DIM. | | | | | | |
