# Supplementary material for: Effects of parity, blood progesterone, and non-steroidal anti-inflammatory treatment on the dynamics of the uterine microbiota of healthy postpartum dairy cows
Source: PLoS One. 2021 Feb 19;16(2):e0233943. doi: 10.1371/journal.pone.0233943 (PMC7895344; doi:10.1371/journal.pone.0233943)
Supplement: S2 Table — (DOCX) [file pone.0233943.s012.docx]

| **S2 Table.** Endometrial polymorphonuclear proportions (PMN%) in clinically healthy postpartum Holstein cows from which endometrial cytobrush samples were collected at 10, 21, and 35 d in milk (DIM) to study their uterine microbiome. | | | | | |
| --- | --- | --- | --- | --- | --- |
| **Group^1^** | | **10 DIM PMN%** | **21 DIM PMN%** | **35 DIM PMN%** | **> 5% PMN at 35 DIM** |
| **Treatment** | **CON** (n = 9) | 44.1 ± 33.6 | 34.2 ± 17.5 | 9.7 ± 8.2 | 55.5% (n = 5) |
|  | **MEL** (n = 7) | 38.7 ± 17.6 | 28.2 ± 19.8 | 10.7 ± 12.4 | 42.8% (n = 3) |
| **Parity** | **PRIM** (n = 7) | 34.5 ± 17.7 | 30.1 ± 21.3 | 10.2 ± 11.1 | 42.8% (n = 3) |
|  | **MULT** (n = 9) | 47.4 ± 32.5 | 32.8 ± 16.5 | 10.1 ± 9.6 | 55.5% (n = 5) |
| **Progesterone** | **LOW** (n = 6) | 47.5 ± 28.9 | 29.3 ± 20 | 12.5 ± 12.6 | 50% (n = 3) |
|  | **HIGH** (n = 10) | 38.3 ± 26.9 | 33.1 ± 17.9 | 8.8 ± 8.3 | 50% (n = 5) |
| ^1^CON, control cows; MEL, cows received meloxicam (MEL, 0.5 mg/kg SC, n = 11) once daily for 4 d (10-13 (DIM)); PRIM, primiparous cows; MULT; multiparous cows; LOW, ≤ 1 ng/mL blood progesterone concentration at 35 DIM ; HIGH, ˃ 1 ng/mL blood progesterone concentration at 35 DIM. | | | | | |
